# SARS-CoV-2 Circulation in the School Setting: A Systematic Review and Meta-Analysis

**DOI:** 10.3390/ijerph19095384

**Published:** 2022-04-28

**Authors:** Saverio Caini, Chiara Martinoli, Carlo La Vecchia, Sara Raimondi, Federica Bellerba, Oriana D’Ecclesiis, Clementina Sasso, Alessandra Basso, Giulio Cammarata, Sara Gandini

**Affiliations:** 1 Institute for Cancer Research, Prevention, and Clinical Network (ISPRO), Via Cosimo il Vecchio 2, 50139 Florence, Italy; s.caini@ispro.toscana.it; 2 Department of Experimental Oncology, European Institute of Oncology (IEO), IRCCS, Via Giuseppe Ripamonti 435, 20141 Milan, Italy; martinoli.chiara@gmail.com (C.M.); sara.raimondi@ieo.it (S.R.); federica.bellerba@ieo.it (F.B.); oriana.decclesiis@ieo.it (O.D.); giulio.cammarata@ieo.it (G.C.); 3 Department of Clinical Sciences and Community Health, Università Degli Studi di Milano, 20133 Milan, Italy; carlo.lavecchia@unimi.it; 4 The Italian National Institute for Astrophysics (INAF)-Osservatorio Astronomico di Capodimonte, Salita Moiariello 16, 80131 Naples, Italy; clementina.sasso@inaf.it; 5 Centre for Philosophy of Social Science (TINT), Unit of Practical Philosophy, Department of Political and Economic Studies, University of Helsinki, P.O. Box 24, 00014 Helsinki, Finland; alessandra.basso@helsinki.fi

**Keywords:** SARS-CoV-2, infections, schools, students, teachers, susceptibility, contract tracing, meta-analysis, screening

## Abstract

The contribution of children to viral spread in schools is still debated. We conducted a systematic review and meta-analysis of studies to investigate SARS-CoV-2 transmission in the school setting. Literature searches on 15 May 2021 yielded a total of 1088 publications, including screening, contact tracing, and seroprevalence studies. MOOSE guidelines were followed, and data were analyzed using random-effects models. From screening studies involving more than 120,000 subjects, we estimated 0.31% (95% confidence interval (CI) 0.05–0.81) SARS-CoV-2 point prevalence in schools. Contact tracing studies, involving a total of 112,622 contacts of children and adults, showed that onward viral transmission was limited (2.54%, 95% CI 0.76–5.31). Young index cases were found to be 74% significantly less likely than adults to favor viral spread (odds ratio (OR) 0.26, 95% CI 0.11–0.63) and less susceptible to infection (OR 0.60; 95% CI 0.25–1.47). Lastly, from seroprevalence studies, with a total of 17,879 subjects involved, we estimated that children were 43% significantly less likely than adults to test positive for antibodies (OR 0.57, 95% CI 0.49–0.68). These findings may not applied to the Omicron phase, we further planned a randomized controlled trial to verify these results.

## 1. Introduction

The global public health crisis due to the severe acute respiratory syndrome coronavirus 2 (SARS-CoV-2) pandemic has brought distinct challenges to the care of children and adolescents. School closures have been implemented internationally as a common strategy to control the spread of SARS-CoV-2 during the pandemic on the basis of the assumption that children may represent important vectors for viral spread. According to UNESCO, 188 countries have imposed countrywide school closures, affecting more than 1.5 billion children and youth, and schooling has been disrupted for an average of 25 weeks worldwide from the beginning of the pandemic until March 2021, due to complete or partial closures (www.unesco.org (accessed on 12 March 2022)). The consequences of school closures could be dramatic. It has been estimated that over 100 million children will fall below the minimum proficiency level in reading due to the impact of COVID-19 school closures (www.unesco.org, accessed on 12 March 2022), and children with disabilities and special needs or those living in countries or areas with poor digital connectivity are especially hard to serve through remote schooling. Beyond providing instruction, school plays a pivotal role in child education, development, and wellbeing. According to UN reports, over 300 million children rely on school meals for a regular source of daily nutrition, and rising malnutrition is expected among the most vulnerable. Lockdowns and shelter-in-place measures have heightened the risk of children witnessing or suffering domestic violence and abuse. Use of online platforms for distance learning has the increased risk of exposure to inappropriate content and online predators, while risks to child mental health and wellbeing are also considerable (www.un.org, accessed on 12 March 2022). 

Although data collected from contact tracing and population studies have indicated that children and adolescents are less susceptible to SARS-CoV-2 infection when compared to adults, as shown in a recent meta-analysis [1], the contribution of children to viral spread is still under debate. In fact, given the typically mild clinical course of COVID-19 in younger age [2,3], symptom-based testing may have underestimated infection in children, and unrecognized viral circulation may still occur at school, potentially raising community infection rates. 

Schools provide a highly regulated environment which is well suited to the investigation of potential COVID-19 exposure [4,5,6,7]. 

The objective of this study was to carry out a comprehensive review of the literature and a meta-analysis covering the evidence on SARS-CoV-2 transmission in the educational setting, collecting data from publications on the prevalence of SARS-CoV-2 positivity, serosurveys, and contact tracing studies. In particular the aims were to estimate SARS-CoV-2 infections detected in schools comparing positive rates found from screening and contact tracing methods, to compare infectivity and susceptibility of students compared to school staff, and to investigate reasons of between-study heterogeneity.

## 2. Materials and Methods

### 2.1. Search Strategy and Selection Criteria

We included any original study (article, communication, report), peer-reviewed publication, or pre-print which reported a quantitative estimation of SARS-CoV-2 transmission in the school setting. We excluded reviews, meta-analyses, and modeling studies. Studies performed in special settings were included in the systematic review but excluded from the meta-analysis. We planned and conducted a systematic literature search and review following MOOSE guidelines regarding the meta-analysis of observational studies. 

**Figure 1:**
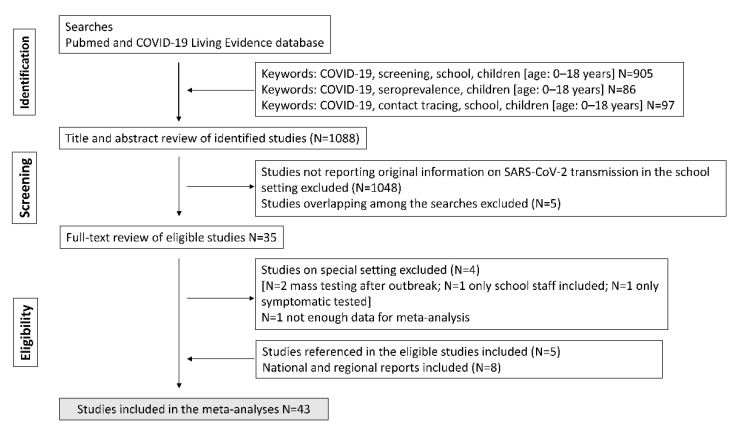
Flowchart of study.

We performed a systematic literature review using validated search strategies from the following databases: PUBMED (http://www.ncbi.nlm.nih.gov/entrez/query.fcgi, accessed on 15 May 2021), Ovid MEDLINE database, ISI Web of Science: Science Citation Index Expanded (SCI Expanded), and Living Evidence on COVID database (https://zika.ispm.unibe.ch/assets/data/pub/search_beta/, accessed on 15 May 2021), to identify papers on SARS-CoV-2 transmission in the educational setting (see flowchart in Figure 1). 

The search was undertaken on 15 May 2021. We used the search terms “screening (or point prevalence)”, “serosurvey (or seroprevalence)”, and “contact tracing” combined with “school (or education), children”. For PUBMED searches, we also limited the search to an age of birth–18 years and added the term “COVID (or COVID-19, or SARS-CoV-2)”. No language restriction was applied. The searches yielded a total of 1088 publications. After screening of titles and abstracts, as well as the removal of duplicates, 35 publications were selected for full-text review. After full-text review, six studies were deemed not eligible for the meta-analysis, and five additional studies were identified after a search of cited references in eligible publications. Finally, seven national and regional reports SARS-CoV-2 transmission in the educational setting fulfilling inclusion criteria were identified and included in the meta-analysis. 

Data were extracted and cross-checked independently by two investigators. The following information from the published papers was extracted and coded: publication type, country, size of the country, region, study period, study setting, study design, exposure to SARS-CoV-2, test method, test sample, number of subjects tested and testing positive in each category (students or staff), and proportions of positive subjects with 95% confidence interval (CI) and confounders adjusted for. In addition, number, age, and definition of index cases and their contacts were collected for contact tracing studies.

This study followed the Preferred Reporting Items for Systematic Reviews and Meta-analyses (PRISMA) reporting guideline [8]. Methodological quality of included studies was assessed on the basis of a critical appraisal checklist for prevalence studies (The Joanna Briggs Institute Critical Appraisal tools for use in JBI Systematic Reviews).

### 2.2. Data Analysis

Inclusion criteria were as follows: (1) the study contains the minimum information necessary to obtain the percentages of positive subjects, or data to calculate risk estimates, by type of index case, and corresponding 95% CI (i.e., odds ratios or relative risks and a measure of uncertainty: standard errors, variance, confidence intervals, or exact *p*-value of the significance of the estimates); (2) the study is based on independent data to avoid giving double weight to a single study. In case of multiple reports of the same study, we considered the estimates from the most recent publications. 

Pooled estimates of percentages of positivity were obtained through random-effects models after Freeman–Tukey double arcsine transformation. In scenarios with zero cases, Haldane–Anscombe correction was used. All measures of association and the corresponding CI were translated into log relative risk and corresponding variance, using the formula proposed by Greenland et al. [9]. We used random-effects models, taking into account between-study and within-study variability when more than one estimate from a single study was used. The summary odds ratio (SOR) was obtained from the maximum likelihood estimate PROC MIXED in SAS, taking into account the model when more estimates were obtained from a single study. Homogeneity of effects across studies was assessed using the chi-square statistic and quantified by I^2^, which represents the percentage of total variation across studies that is attributable to heterogeneity rather than chance [10]. We obtained the SOR pooling the study-specific estimates through random-effects models. A funnel-plot-based approach was used for assessing publication bias evaluating regression of log(OR) on the sample size, weighted by the inverse of the variance, as suggested by Macaskill et al. [11]. 

To assess the influence of possible sources of bias, we considered the STROBE (Strengthening the Reporting of Observational Studies in Epidemiology) checklist proposed for observational epidemiologic studies [12]. According to the STROBE checklist, using meta-regression, we evaluated factors influencing between-study heterogeneity. We also examine changes in results after exclusion of specific studies to evaluate the stability of the pooled estimates. Sensitivity analysis was carried out to evaluate whether results were influenced by single studies.

All the statistical analyses were performed using SAS software (SAS Institute Inc., Cary, NC, USA; version 9.4) and R software, version 4.0.2 (http://www.r-project.org, accessed on 12 March 2022). Two-sided *p*-values less than 0.05 were considered statistically significant. 

## 3. Results

### 3.1. SARS-CoV-2 Positivity Rates in Educational Settings (Screening Studies)

**Figure 2:**
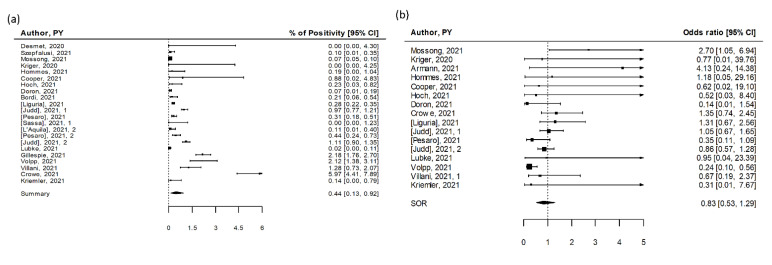
Pooled estimates for SARS-CoV-2 screening studies [13,14,15,16,17,18,19,20,21,22,23,24,25,26,27,28,29,30,31,32,33]. (**a**) Pooled estimate of testing positive for SARS-CoV-2. (**b**) Summary odds ratio of testing positive for SARS-CoV-2 for children versus adults linked to educational settings.

We identified 22 studies that reported data on SARS-CoV-2 infections in diverse educational settings, from kindergartens and daycares to primary and high schools (Table S1 in the Supplementary Materials) [13,14,15,16,17,18,19,20,21,22,23,24,25,26,27,28,29,30,31,32,33], involving more than 120,000 subjects. Sixteen studies were from Europe, five were from the US, and one was from Israel. Overall, studies documented SARS-CoV-2 positivity rates from the beginning of the pandemic in March 2020 until May 2021, including periods of low and high community transmission. Screening campaigns were organized by schools as part of mitigation measures to prevent the introduction of SARS-CoV-2 in their premises or by local and national authorities to monitor viral circulation among students, teachers, and nonteaching staff. Testing involved asymptomatic or oligosymptomatic participants, providing an indication of otherwise possibly unrecognized viral spread within educational settings. The vast majority of the studies were judged to be of high quality (80%, Table S4). The summary estimate of positivity rate assessed through implementation of the different screening methods was 0.44% (95% CI 0.13–0.92%), with high heterogeneity (I^2^ = 97%, Figure 2a). The difference in estimates between cross-sectional and cohort studies was significant (*p* = 0.03) with a remarkably lower prevalence among cross-sectional studies (0.31%, 95% CI 0.05–0.81%) compared to cohort studies (1.14%, 95% CI 0.01–4.19%) (Table 1). 

Sixteen studies reported SARS-CoV-2 point prevalence in a total of 112,131 subjects, including one study providing data collected during two rounds of testing within the COVID-19 Schools Infection Survey. A total of 326 coronavirus infections were detected. Overall, the estimated positivity rate was 0.31% (95% CI 0.05–0.81%), with high heterogeneity among studies (I^2^ = 95%, Table 1). Highest infections rates were reported in the two rounds of testing performed in England in November and December 2020, especially among high school students and staff. Excluding reports not published in peer-reviewed journals by April 2021, the summary estimate of positivity rate was slightly greater, 0.40% (95% CI 0.05–1.07%), but still below 1%.

Six cohort studies reported results of multiple testing performed on a total of 12,838 subjects. Multiple testing, especially when intensive schedules over a prolonged period are implemented, may provide indications on the cumulative viral spread within the analyzed educational settings. Overall, estimated positivity rate was 1.14% (95% CI 0.01–4.19%), with high heterogeneity among studies (I^2^ = 98%, Table 1). The highest number of cases was detected in the study of Crowe and colleagues [28], who identified 46 infections in 773 asymptomatic staff and students (5.6%) from three schools engaged in a pilot program with weekly testing over a 5 week period in November 2020 in Omaha, US. Biweekly testing led to the identification of 25 cases out of 1180 participants (2.1%) over 3 month period in the study by Volpp and colleagues in New Jersey, US (15–22 samples collected on average from each participant, for a total of 21,449 test performed) [20], while only one case out of 5210 participants (0.02%) was detected in a 4 week study performed in Dusseldorf, Germany (34.068 tested samples, up to eight tests per subject) [17]. Two rounds of testing 1 week apart identified one case out of 707 participants in Swiss students and staff participating to Ciao corona study [29], while 16 samples tested positive out of the 3431 collected from 1251 students and staff (1.3% positive subjects) of two schools in Rome, Italy, over a 3 month period [23]. Lastly, Gillespie and colleagues reported the experience of two US schools that implemented strong mitigation measures for reopening [19], including weekly testing for students and staff. Here, nine rounds of universal testing led to the identification of 81 SARS-CoV-2-infected individuals out of the 3720 participants (2.2%), with an additional 23 infections identified through contact tracing and 33 self-reported COVID-19 cases. 

Nine cross-sectional studies and five cohort studies reported infections detected in children and in adults separately, including one reporting estimates for two rounds of testing (Table S1 in the Supplementary Materials). Children and adults showed comparable SARS-CoV-2 positivity rates in most studies, and the pooled OR estimate was 0.83 (95% CI: 0.53–1.29), with low heterogeneity (I^2^ = 41%, Figure 2b). We found an indication for publication bias (*p* = 0.03). Similar results were observed in cross-sectional studies (pooled OR = 0.98; 95% CI 0.74–1.32, I^2^ = 23%) and cohort studies (pooled OR = 0.62; 95% CI 0.20–1.94, I^2^ = 4%) (Table 1).

### 3.2. SARS-CoV-2 Seroprevalence in Educational Settings (Serosurveys)

**Figure 3:**
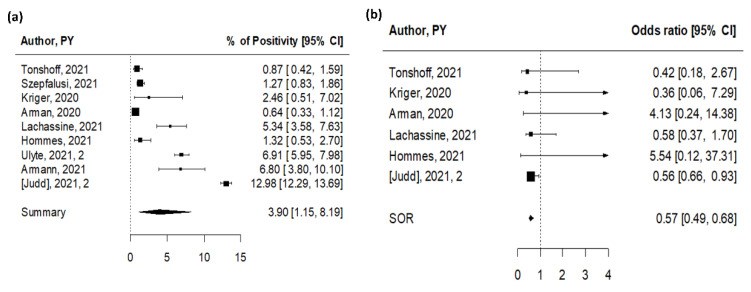
Pooled estimates for SARS-CoV-2 seroprevalence studies [14,16,18,27,34,35,36,37,38]. (**a**) Pooled estimate of testing positive for antibodies for SARS-CoV-2. (**b**) Pooled estimate of odds of testing positive for antibodies for SARS-CoV-2 for children versus adults.

We identified nine studies that reported data on SARS-CoV-2 seroprevalence in educational settings providing in-person activities [14,16,18,27,34,35,36,37,38], with a total of 17,879 subjects involved (Table S2 in the Supplementary Materials). All studies were performed in Europe during 2020. While the above-described point prevalence cross-sectional studies inform on the rates of current infections, cross-sectional serosurveys inform on cumulative exposure to the virus for tested participants. We also identified three cohort studies assessing prevalence of antibodies for SARS-CoV-2 at different timepoints, with participants tested twice to examine longitudinal changes of seroprevalence [27,37,38], and we included the most recent testing in our metanalysis. The majority of the studies were judged to be of high quality (70%, Table S5). The overall seropositive rate was 3.9% (95% CI 1.15–8.19%), with high heterogeneity among studies (I^2^ = 100%, Figure 3a). The difference in estimates between cross-sectional and cohort studies was statistically significant (*p* = 0.005), with estimates obtained from cohort studies indicating 10% positivity (10.31%; 95% CI 2.44–22.74%), compared to a lower prevalence of 1.5% from cross-sectional studies (1.49%, 95% CI 0.07–4.69%) (Table 1). 

Six studies were performed during the first semester of 2020, with antibodies assessed during or shortly after the first wave of the pandemic, including three studies focused on attendance to schools and daycares that remained exceptionally open during national lockdowns. Although the seroprevalence differed among the three studies, authors reported comparable seroprevalence in groups of children not attending school (0.5% versus 1% and 1.4% versus 2.7%, respectively, for children attending in person or staying at home), as well as in adults who did not have occupational contact with children or COVID-19-positive patients (7.7% for daycare staff and 5.5% for the comparator adult group), suggesting that exceptional schooling did not boost SARS-CoV-2 spread in the analyzed settings. In line with these observations, low seroprevalence reported in three additional cross-sectional studies adds up to the indication that schools did not develop into silent hotspots for viral transmission during the first wave of the pandemic [14,18,35], likely due to the successful implementation of extensive preventive measures. In this regard, it is noteworthy to mention two studies reporting seroprevalence in students from a small city in north France and from a large community school in Santiago, Chile [39,40]. In both cases, school-based COVID-19 outbreaks occurred at the very onset of the pandemic and in the absence of preventive measures, leading to 38.1% and 43.4% seropositive pupils and teachers, respectively, in one French high school [39] and 9.9% and 16.6% seropositive students and staff in the Chilean study [40]. 

In the three cohort studies [27,37,38], increased seroprevalence was recorded during the second study visit, which took place after the summer break or in December 2020. In their study, Ulyte and colleagues reported that SARS-CoV-2 seroprevalence raised from 3% in the summer to 4.5% in late autumn in school children in the canton of Zurich, Switzerland [37]. Interestingly, among the children who participated in both testing rounds, 28/70 (40%) who were initially seropositive became seronegative, while seroconversion (previously seronegative participants who became seropositive) was 5% (109/2153). The estimated rate of ever-positive children was, therefore, 7.8% [37]. Serial blood sampling was also implemented in a secondary school in Dresden, Germany [38]. Here, antibody positivity rates increased from 1.7% to 6.8% during the 6 week study period, and all the participants who tested positive at the initial visit (5/5) remained positive at the second visit. Lastly, data collected within the COVID-19 Schools Infection Survey showed high initial antibody positivity rates, consistent with the study designed to oversample schools in areas of England where coronavirus infection was highest at the start of the academic year (September 2020), with a nonsignificant increase in pupils (7.7% to 9% and 11% to 13.5% for primary and secondary school, respectively) and staff (12.5% to 15%) testing positive for SARS-CoV-2 antibodies between November and December 2020. In this study, 7.3% (20/276) of staff who initially tested positive had no detectable antibodies in the second round. 

Separate seroprevalence estimates for children and adults were available for six studies (Table S2 in the Supplementary Materials) [16,18,27,34,35,36]. School-aged children had lower antibody positivity rates when compared with adults (parents or school staff) in 4/6 studies (Figure 3b). The pooled OR for children was 0.57 (95% CI 0.49–0.68), significantly lower than adults, with low heterogeneity among studies (I^2^ = 21%). No indication of publication bias was found (*p* = 0.42). 

### 3.3. Onward Transmission of SARS-CoV-2 in Educational Settings (Contact Tracing)

Studies analyzing onward transmission of SARS-CoV-2 offered the possibility to test more specifically the infectivity and susceptibility to infection of children linked to educational settings. We identified 15 studies that reported data on transmission of SARS-CoV-2 in schools with available information on the number of contacts of index cases (Table S3 in the Supplementary Materials) [16,41,42,43,44,45,46,47,48,49,50,51,52,53,54]. Six studies were from Europe, five were from the USA, and two were from Israel, along with one each from South Korea and Australia. A total of 112,622 contacts of children and adults who were physically present at school while positive for SARS-CoV-2 were identified. Molecular testing for SARS-CoV-2 was normally offered to all contacts exposed to SARS-CoV-2, except for one study [48]. In this study, only symptomatic contacts were tested, so asymptomatic secondary cases were not captured. The majority of the studies were judged to be of high quality (60%, Table S6).

**Figure 4:**
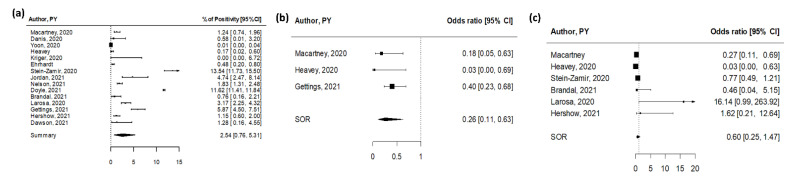
Pooled estimates for contact tracing studies [16,41,42,43,44,45,46,47,48,49,50,51,52,53,54]. (**a**) Pooled estimate of testing positive for SARS-CoV-2 after a contact with a case in the school setting. (**b**) Summary OR of testing positive for SARS-CoV-2 after a contact with a young versus an adult case in the school setting. (**c**) Summary OR of testing positive for SARS-CoV-2 in young versus adults after a contact with a case.

When considering any-age index cases and their contacts of any age, the pooled secondary attack rate (SAR) was 2.54% (95% CI 0.76–5.31%), with high heterogeneity among studies (I^2^ = 100%, Figure 4a). The highest attack rate (13.5%) was recorded within a large outbreak in a high school in Jerusalem, Israel linked to two student index cases and likely promoted by inadequate preventive measures (crowded classes, exemption from facemasks, and continuous air conditioning due to an extreme heatwave) [46]. High attack rates were also recorded in two US-based studies, both reporting transmission with index cases of any age. Doyle and colleagues investigated COVID-19 in primary and secondary schools in Florida during the first semester of school reopening [49]. Of the 63,654 of COVID-19 cases registered between August and December 2020 in school-aged children, 60% were not school-related, and <1% of registered students were identified as having school-related COVID-19. Contact tracing investigations identified 86,832 persons who had a close school contact with these cases; among these, 37,548 received testing and 10,092 received a positive SARS-CoV-2 test result, leading to a 11.6% secondary attack rate. A prospective investigation of SARS-CoV-2 transmission was also performed in a Georgia school district during a period of peak community COVID-19 incidence [55]. Tracing of 86 index cases identified 1005 contacts, of whom 644 were tested and 59 received a positive SARS-CoV-2 test result (SAR = 5.9%). Highest SARs were identified in the setting of indoor sports and staff interactions. On the other hand, extremely low attack rates (<1%) were found in five studies reporting from five nations and two continents and describing transmission of SARS-CoV-2 in a school setting from the early onset of the pandemic (one pediatric case among the first reported cases in France who visited three schools and one ski class while infected generated 172 contacts and one secondary case) until November 2020 [42]. 

Three studies reported on viral transmission from young (age < 18 years) or adult index cases (Table S3 in the Supplementary Materials) [41,44,52]. In all studies, the proportion of contacts infected by young index cases was lower compared to adult index cases (Figure 4b). The pooled OR was 0.26 (95% CI 0.11–0.63), indicating a significant threefold reduced infectivity for children compared with adults. No indication for publication bias was found (*p* = 0.59). 

Six studies reported estimates of viral transmission to young or adult contacts (Table S3 in the Supplementary Materials) [41,44,46,50,51,53]. The pooled OR was 0.60 (95% CI 0.25–1.47), indicating a not statistically significant reduced susceptibility to SARS-CoV-2 infection for children compared to adults (Figure 4c). No indication of publication bias was found (*p* = 0.65). 

## 4. Discussion

The findings of this systematic review and meta-analysis confirm that schools did not develop into hotspots for SARS-CoV-2 transmission, as already emerged from the contact tracing study by Gandini et al. [55], which did not support the hypothesis of school openings as a driver of the second wave of COVID-19 in Italy, a large European country with high incidence of SARS-CoV-2. Likewise, another Italian study analyzed SARS-CoV-2 seroprevalence among school-aged children between September 2020 and January 2021 in Milan. The overall seroconversion rate was 10%, with no differences found between students who attended school compared to those who started remote learning in the first days of November, and most patients (61%) reporting that contact with a confirmed COVID-19 patient occurred within the household. The authors concluded that schools did not amplify SARS-CoV-2 transmission, but rather mirrored the level of the transmission in the community [56]. A Japanese study published in Nature also concluded that there was no causal effect of school closures on the spread of SARS-CoV-2 in spring 2020 [57]. Recent publications are in agreement with results found in this meta-analysis showing that students are less susceptible and less infective, with a large Italian study showing that secondary infections occurred more frequently when the index case was a teacher than a student (37% vs. 10%, *p* = 0.007) [55]. Bark et al. [58] assessed infections in kindergartens in Canada and found that school-based transmissions of SARS-CoV-2 were rare and clusters were small. Staff members accounted for 53.8% of index cases even if they were 14.3% of the school population. A recent Swedish study confirmed that keeping lower-secondary schools open had a minor impact on the overall spread of SARS-CoV-2 in society despite a twofold increase in infection rate found in teachers compared to students [59]. A study carried out in Australia showed that the majority of events (66%) had no evidence of onward transmission. Furthermore, when outbreaks did occur, they were mostly small (<10 cases) and more common when the first case was an adult (age > 16) [60]. A recent study conducted in USA investigated differences among three waves and among different working places, finding that schools experienced 11% of identified outbreaks, yet involved just 4% of total cases, whereas adult education outbreaks (2%) accounted for disproportionately more cases (9%). The authors concluded that schools were not the key driver of the latest wave in infections [61]. Some studies found an increase in infections with opening of schools, but they are mainly modeling studies [62,63]. Although infection can and does occur in schools, modeling studies have indicated limited viral spread when mitigation measures are adopted [64]. In particular, one modeling study concluded that weekly testing of 75% of unvaccinated students, in addition to symptom-based testing, would reduce cases by about 35% compared with symptom-based testing alone. Regular testing would also reduce student-days lost by up to 80% compared with reactive class closures [65].

Reviewing the experience of different schools showed that health behavioral policies adopted before the vaccination helped in mitigating the risk of viral spread in educational settings. In particular, contact tracing was very useful to promptly isolate infected staff and students. We found fivefold greater frequency of positive tests with contact tracing compared to screening, and these results suggests that we need a randomized clinical trial to verify whether testing all subjects in schools, independently of symptoms, helps in reducing clusters. 

Engagement of all stakeholders (school staff members, students, and their parents or legal guardians) in the implementation of school-based policies, as well as individual adherence to shared recommendations, is essential for minimizing the COVID-19 transmission chain. 

Many governments have ordered school closures to prevent contagions. Numerous studies have shown that this decision had a negative impact not only on the learning loss [66] in students but also on their mental health [67,68]. In particular, it appears that the students most affected were those who were younger and from families with low socioeconomic status [69]. Our meta-analysis could be a tool to help balance the pros and cons of school closures and eliminate the inequities that the pandemic has brought. 

There are some limitations of this study to highlight. Firstly, the risk estimates of each study were calculated from the minimum necessary information in the respective articles included, such that not all possible confounding factors could be taken into account and the risk estimates were not fully adjusted. Secondly, there was significant between-study heterogeneity and publication bias in some scenarios. Thirdly, the majority of the articles analyzed presented data from observational studies with some potential sources of bias. However this is the first meta-analysis investigating all sources of between-study heterogeneity, study designs, countries, methods adopted to investigate infections, and age to be able to have information about causal associations [70]. 

During the period of conduct of each study, mitigation measures of COVID-19 were put in place, and we cannot exclude that these contributed to the decrease in the spread of the virus in schools. Furthermore, we did not have enough information to quantify the impact of those measures. A recent randomized trial revealed, in line with our findings, low infection rates in school contacts, with a very small number of positive school contacts (around 2%) [71], and daily contact testing of school-based contacts was noninferior to self-isolation for the control of COVID-19 transmission. 

## 5. Conclusions

Testing all subjects in schools, independently of symptoms, revealed that students are less likely than adults to favor viral spread, and SARS-CoV-2 circulation in schools was found to be limited. These findings may not apply to the Omicron phase. We are therefore planned a clinical trial to confirm the results of this meta-analysis (https://eucareresearch.eu/activities/school-studies/the-interventional-lolli-study/, accessed on 12 March 2022). This study, while based on observational studies, presents interesting hypotheses to encourage the planning and conduct of randomized controlled experiments.

## Figures and Tables

**Table 1:** Summary estimates.

		*n*	Summary	Low 95% CI	Up 95% CI	I^2^ (%)	Study Design	*p*-Value
% SARS-CoV-2 positivity	Contract tracing *	15	2.54	0.76	5.31	100		
	Screening	22	0.44	0.13	0.92	97		
		6	1.14	0.01	4.19	98	Cohorts	0.03
		16	0.31	0.05	0.81	95	Cross-sectionals	
	Serosurvey	9	3.90	1.15	8.19	100		
		3	10.31	2.44	22.74	98	Cohorts	0.005
		6	1.49	0.07	4.69	88	Cross-sectionals	
OR for young vs. old	Susceptibility in contract tracing	6	0.60	0.25	1.47	63		
	Infectivity in contract tracing	3	0.26	0.11	0.63	44		
	Screening	15	0.83	0.53	1.29	41		
		5	0.62	0.20	1.94	69	Cohorts	0.56
		10	0.98	0.74	1.32	23	Cross-sectionals	
	Serosurvey	6	0.57	0.46	0.68	21		

* Evaluated considering contacts as denominators instead of screened subjects; *p*-value from meta-regression for study design. OR: Odd Ratios.

## Data Availability

Not applicable as this is a review and meta-analysis of published data.

## Supplementary Materials

The following supporting information can be downloaded at https://www.mdpi.com/article/10.3390/ijerph19095384/s1: Table S1. Studies on screening for SARS-CoV-2 infections; Table S2. Studies on serosurveys for antibodies to SARS-CoV-2; Table S3. Studies on contact tracing; Table S4. Studies on screening for SARS-CoV-2 infections: quality evaluation; Table S5. Studies on serosurveys for antibodies to SARS-CoV-2: quality evaluation; Table S6. Studies on contact tracing: quality evaluation.
