# Supplementary material for: SARS-CoV-2 Circulation in the School Setting: A Systematic Review and Meta-Analysis
Source: Int J Environ Res Public Health. 2022 Apr 28;19(9):5384. doi: 10.3390/ijerph19095384 (PMC9099553; doi:10.3390/ijerph19095384)
Supplement: Supplementary file 1 [file ijerph-19-05384-s001.zip › ijerph-1657288-supplementary.pdf]

Table S1: Studies on screening for SARS-CoV-2 infections

First author [report]	Country	Study design	Start obs	Stop obs	Test method	Test sample	Subjects tested	N tested	N pos
Desmet[13]	Belgium	Cross-sectional	02/03/2020	12/03/2020	RT-PCR	Swab	Children (Daycare 6-30 mo)	84	0
Szépfalusi[14]	Austria	Cross-sectional	01/05/2020	30/07/2020	RT-PCR	Swab	Students (5-21y)	2064	2
Mossong[15]	Luxembourg	Cross-sectional	04/05/2020	25/07/2020	RT-PCR	Swab	Students and staff<br>Students<br>Staff	48380<br>33723<br>14657	36<br>31<br>5
Kriger[16]	Israel	Cross-sectional	07/05/2020	17/05/2020	RT-PCR	Swab	Children and parents<br>Daycare (8±2.5 y)<br>Parents	85<br>48<br>37	0<br>0<br>0
Lübke[17]	Germany	Cohort	10/06/2020	07/07/2020	RT-PCR	Saliva	Children and staff<br>Daycare (0-7y)<br>Staff	5210<br>3955<br>1255	1<br>1<br>0
Hommes[18]	Germany	Cross-sectional	11/06/2020	19/06/2020	RT-PCR	Swab	Students and staff<br>Primary school students<br>High school students<br>Staff	532<br>193<br>192<br>150	1<br>0<br>1<br>0
Gillespie*[19]	USA	Cohort	5/8/2020	20/12/2020	RT-PCR	Swab or saliva	Students and staff K12 Schools	3720	81*
Volpp[20]	USA	Cohort	20/08/2020	27/11/2020	RT-PCR	Swab	Students and staff<br>Students<br>Staff	1180<br>775<br>405	25<br>8<br>17
Cooper[21]	USA	Cross-sectional	01/09/2020	01/11/2020	RT-PCR	Swab	Students and staff<br>K12 Students<br>Staff	113<br>86<br>27	1<br>1<br>0
Hoch[22]	Germany	Cross-sectional	07/09/2020	30/11/2020	RT-PCR	Saliva and swab	Students and staff<br>Students (3-12y)<br>Staff	875<br>574<br>301	2<br>1<br>1
Villani[23]	Italy	Cohort	21/09/2020	04/12/2020	RT-PCR	Saliva	Students and staff<br>Students<br>Staff	1251<br>1083<br>168	16<br>13<br>3
Doron[24]	USA	Cross-sectional	01/10/2020	08/10/2020	RT-PCR	Saliva	Students and staff<br>K12 students<br>Staff	4601<br>3596<br>1005	3<br>1<br>2
Bordi[25]	Italy	Cross-sectional	06/10/2020	02/11/2020	RT-PCR	Saliva	Students 2-15y	1905	4
[Liguria][26]	Italy	Cross-sectional	09/10/2020	14/05/2021	RDT	Swab	Students and staff<br>Primary school students	25961<br>15191	72<br>44

							High school students<br>Staff	6237<br>4533	18<br>10
[Judd][27]	England	Cross-sectional (Round 1)	03/11/2020	20/11/2020	RT-PCR	Swab	Students and staff<br>Primary school students<br>High school students<br>Staff	7923<br>1936<br>2420<br>3567	.
Crowe[28]	USA	Cohort	09/11/2020	11/12/2020	RT-PCR	Saliva	Students and staff<br>K12 Students<br>Staff	770<br>315<br>455	46<br>22<br>24
[Judd][27]	England	Cross-sectional (Round 2)	30/11/2020	11/12/2020	RT-PCR	Swab	Students and staff<br>Primary school students<br>High school students<br>Staff	8650<br>1898<br>2988<br>3764	.
Kriemler[29]	Switzerland	Cohort	01/12/2020	11/12/2020	RT-PCR	Swab	Students and staff<br>Students (9-14y)<br>Staff	707<br>641<br>66	1<br>1<br>0
[Pesaro][30]	Italy	Cross-sectional	28/01/2021	03/02/2021	RDT	.	Students and staff<br>High school students<br>Staff	5104<br>4568<br>536	16<br>12<br>4
[L'Aquila][31]	Italy	Cross-sectional	19/02/2021	20/02/2021	RDT	Swab	Students and staff	1800	2
[Pesaro][32]	Italy	Cross-sectional	04/03/2021	12/03/2021	RDT	Swab	Students and staff	3200	14
[Sassa][33]	Italy	Cross-sectional	18/05/2021	18/05/2021	RDT	Swab	Students and staff	299	0

*Additional school cases: 23 contact tracing, 33 self-reported

Table S2. Studies on serosurveys for antibodies to SARS-CoV-2

First author [report]	Country	Study design	Start obs	Stop obs	Test method	Test sample	Subjects tested	N tested	N pos
Tönshoff[34]	Germany	Cross-sectional	22/04/2020	15/05/2020	Quantitative	Serum	Students and parents<br>Students<br>Parents	1156<br>580<br>576	10<br>3<br>7
Szépfalusi[14]	Austria	Cross-sectional	01/05/2020	30/07/2020	Quantitative	Serum	Students (5-21y)	2042	26
Kriger[16]	Israel	Cross-sectional	07/05/2020	17/05/2020	Quantitative	Serum	Children and parents<br>Daycare (8±2.5 y)<br>Parents	122<br>70<br>52	3<br>1<br>2
Armann[35]*	Germany	Cross-sectional	25/05/2020	30/06/2020	Quantitative	Serum	Students and staff<br>High school students<br>Staff	2045<br>1538<br>507	12<br>11<br>1
Lachassinne[36]	France	Cross-sectional	04/06/2020	03/07/2020	Qualitative	Finger prick blood	Children and staff<br>Daycare (0-5 y)<br>Parents	524<br>327<br>197	28<br>14<br>14
Hommes[18]	Germany	Cross-sectional	11/06/2020	19/06/2020	Quantitative	Finger prick blood	Students and staff<br>Students<br>Staff	531<br>382<br>149	7<br>7<br>0
Ulyte[37]	Switzerland	Cohort_T1	16/07/2020	09/07/2020	Quantitative	Serum	Students	2496	74
Ulyte[37]	Switzerland	Cohort_T2	26/10/2020	18/11/2020	Quantitative	Serum	Students	2503	173
[Judd][27]	England	Cohort_T1	03/11/2020	20/11/2020	Quantitative	Serum	Students and staff<br>Primary school students<br>High school students<br>Staff	7719<br>1996<br>2449<br>3274	.
[Judd][27]	England	Cohort_T2	30/11/2020	11/12/2020	Quantitative	Serum	Students and staff	8899	.

							Primary school students<br>High school students<br>Staff	2152<br>3280<br>3467	
Armann[38]	Germany	Cohort_T1	Nov2020	Nov2020	Quantitative	Serum	Students and staff (High school)	302	5
Armann[38]	Germany	Cohort_T2	Dec2020	Dec2020	Quantitative	Serum	Students and staff (High school)	273	16

*The first author of this manuscript changed in the peer-reviewed publication.

Table S3. Studies on contact tracing

							**All contacts**		**Contacts <18y**		**Adult contacts**	
**First author**	**Country**	**Start obs**	**Stop obs**	**Contacts**	**Age index**	**N index**	**N**	**N pos**	**N**	**N pos**	**N**	**N pos**
Macartney[41]	Australia	25/01/2020	01/05/2020	Community	Any<br><18y<br>Adult	27<br>12<br>15	1448<br>752<br>696	18<br>3<br>15	.<br>649<br>536	.<br>2<br>8	.<br>103<br>160	.<br>1<br>7
Danis[42]	France	31/01/2020	14/02/2020	In-school	<18y	1	172	1	.	.	.	.
Yoon[43]	South Korea	18/02/2020	31/07/2020	In-school	<18y	44	13100	1	.	.	.	.
Heavey[44]	Ireland	01/03/2020	13/03/2020	Community	Any<br><18y<br>Adult	6<br>3<br>3	1165<br>999<br>166	2<br>0<br>2	.<br>905<br>106	.<br>0<br>0	.<br>94<br>60	.<br>0<br>2
Kriger[16]	Israel	07/05/2020	17/05/2020	In-school	Adult	1	53	0	.	.	53	0
Ehrhardt[45]	Germany	25/05/2020	05/08/2020	In-school	<18y	137	2300	11	.	.	.	.
Stein-Zamir[46]	Israel	28/05/2020	30/05/2020	In-school	<18y	.	1315	178	1164	153	152	25
Jordan[47]	Spain	29/06/2020	31/07/2020	In-school	Any	39	253	12	.	.	.	.
Nelson[48]	USA	01/08/2020	30/11/2020	In-school	<18y	257	2189	40	.	.	2189	40
Doyle[49]	USA	10/08/2020	21/12/2020	Community	Any	.	86832	10092	.	.	.	.
Brandal[50]	Norway	28/08/2020	11/11/2020	In-school	<18y	13	393	3	319	2	74	1
Larosa[51]	Italy	01/09/2020	15/10/2020	In-school	Any	48	1200	38	204	0	996	38
Gettings[52]	USA	01/12/2020	22/01/2021	In-school	Any<br><18y<br>Adult	86<br>53<br>33	1005<br>689<br>421	59<br>24<br>35	.	.	.	.
Hershow[53]	USA	03/12/2020	31/01/2021	In-school	Any	51	1041	12	908	11	133	1
Dawson[54]	USA	07/12/2020	18/12/2020	In-school	Any	37	156	2	.	.	.	.

Table S4: Studies on screening for SARS-CoV-2 infections: quality evaluation

First author	1. Was the sample frame appropriate to address the target population?	2. Were study participants sampled in an appropriate way?	3. Was the sample size adequate?	4. Were the study subjects and the setting described in detail?	5. Was the data analysis conducted with sufficient coverage of the identified sample?	6. Were valid methods used for the identification of the condition?	7. Was the condition measured in a standard, reliable way for all participants?	8. Was there appropriate statistical analysis?	9. Was the response rate adequate, and if not, was the low response rate managed appropriately?	Quality
Desmet[13]	0	1	0	1	0	1	1	0	1	Low
Szépfalusi[14]	1	1	1	1	0	1	1	0	1	High
Mossong[15]	1	1	1	1	1	1	1	0	1	High
Kriger[16]	0	1	0	1	0	1	1	0	1	Low
Lübke[17]	1	1	1	1	1	1	1	1	1	High
Hommes[18]	1	1	0	1	0	1	1	0	1	medium
Gillespie[19]	1	1	1	1	1	1	1	1	1	High
Volpp[20]	1	1	1	1	1	1	1	1	1	High
Cooper[21]	0	1	0	1	0	1	1	0	1	Low
Hoch[22]	1	1	1	1	1	1	1	0	1	High
Villani[23]	1	1	1	1	1	1	1	1	1	High
Doron[24]	1	1	1	1	1	1	1	0	1	High
Bordi[25]	1	1	1	1	0	1	1	0	1	High
[Liguria][26]	1	1	1	1	1	0	1	0	1	High
[Judd][27]	1	1	1	1	1	1	1	0	1	High
Crowe[28]	1	1	1	1	1	1	1	1	1	High
[Judd][27]	1	1	1	1	1	1	1	0	1	High
Kriemler[29]	1	1	1	1	1	1	1	1	1	High
[Pesaro][30]	1	1	1	1	1	0	1	0	1	High

[L'Aquila][31]	1	1	1	1	1	0	1	0	1	High
[Pesaro][32]	1	1	1	1	1	0	1	0	1	High
[Sassa][33]	0	1	0	1	0	0	1	0	1	Low

Table S5: Studies on serosurveys for antibodies to SARS-CoV-2: quality evaluation

**First author**	1. Was the sample frame appropriate to address the target population?	2. Were study participants sampled in an appropriate way?	3. Was the sample size adequate?	4. Were the study subjects and the setting described in detail?	5. Was the data analysis conducted with sufficient coverage of the identified sample?	6. Were valid methods used for the identification of the condition?	7. Was the condition measured in a standard, reliable way for all participants?	8. Was there appropriate statistical analysis?	9. Was the response rate adequate, and if not, was the low response rate managed appropriately?	Quality
Tönshoff[34]	1	1	1	1	1	1	1	0	1	High
Szépfalusi[14]	0	0	1	1	0	1	0	0	1	Low
Kriger[16]	0	0	0	1	0	1	0	0	0	Low
Armann[35*]	1	1	1	1	1	1	1	0	1	High
Lachassinne[36]	1	1	1	1	1	0	1	0	1	High
Hommes[18]	1	1	1	1	1	0	1	0	1	High
Ulyte[37]	0	0	1	1	0	1	0	1	1	Medium
Ulyte[37]	0	0	1	1	0	1	0	1	1	Medium
[Judd][27]	1	1	1	1	1	1	1	1	1	High
[Judd][27]	1	1	1	1	1	1	1	1	1	High
Armann[38]	1	1	0	1	1	1	1	1	0	High
Armann[38]	1	1	0	1	1	1	1	1	0	High

*The first author of this manuscript changed in the peer-reviewed publication.

Table S6: Studies on contact tracing: quality evaluation

**First author**	1. Was the sample frame appropriate to address the target population?	2. Were study participants sampled in an appropriate way?	3. Was the sample size adequate?	4. Were the study subjects and the setting described in detail?	5. Was the data analysis conducted with sufficient coverage of the identified sample?	6. Were valid methods used for the identification of the condition?	7. Was the condition measured in a standard, reliable way for all participants?	8. Was there appropriate statistical analysis?	9. Was the response rate adequate, and if not, was the low response rate managed appropriately?	Quality
Macartney[41]	0	1	1	1	1	0	1	1	1	High
Danis[42]	1	0	0	1	0	1	1	0	1	Medium
Yoon[43]	1	1	1	1	0	1	1	0	1	High
Heavey[44]	0	1	1	1	1	0	1	1	1	High
Kriger[16]	1	0	0	1	0	1	1	0	1	Medium
Ehrhardt[45]	1	1	1	1	0	1	1	0	1	High
Stein-Zamir[46]	1	0	1	1	0	1	1	1	1	High
Jordan[47]	1	1	0	1	1	1	0	0	1	Medium
Nelson[48]	1	1	1	1	0	1	1	0	1	High
Doyle[49]	0	0	1	1	1	0	0	0	1	Low
Brandal[50]	1	0	0		0	1	1	1	1	Medium
Larosa[51]	1	1	1		1	1	0	1	1	High
Gettings[52]	1	1	1	1	1	1	1	0	1	High

Hershow[53]	1	1	1	1	1	1	0	1	1	High
Dawson[54]	1	1	0	1	1	1	0	0	1	Mediu m
